# Early palliative care for solid and blood cancer patients and caregivers: Quantitative and qualitative results of a long-term experience as a case of value-based medicine

**DOI:** 10.3389/fpubh.2023.1092145

**Published:** 2023-03-06

**Authors:** Sarah Bigi, Eleonora Borelli, Leonardo Potenza, Fabio Gilioli, Fabrizio Artioli, Giampiero Porzio, Mario Luppi, Elena Bandieri

**Affiliations:** ^1^Department of Linguistic Sciences and Foreign Literatures, Università Cattolica del Sacro Cuore, Milan, Italy; ^2^Department of Medical and Surgical Sciences, University of Modena and Reggio Emilia, Modena, Italy; ^3^Hematology Unit and Chair, Azienda Ospedaliera Universitaria di Modena, Modena, Italy; ^4^Department of Internal Medicine and Rehabilitation, USL, Modena, Italy; ^5^Oncology and Palliative Care Units, Civil Hospital Carpi, USL, Carpi, Italy; ^6^Tuscany Tumor Association, Florence, Italy

**Keywords:** early palliative care, advanced care, communication, value-based care, cancer

## Abstract

**Introduction:**

Cancer patients and their caregivers have substantial unmet needs, that negatively impact the clinical outcome and quality of life. However, interventions aimed to address such needs are still suboptimal, failing to answer the recent healthcare call for the adoption of value-based models of care. In the case of incurable oncologic and hematologic cancers, a value-based model of care should plan advanced care on patients' needs and include the quality of death as an outcome. The integration of early palliative care into standard oncologic care for patients with advanced cancers represents a recent innovative model of assistance whose benefits for patients and caregivers are now widely recognized. The key elements underlying the reasons behind these benefits are the multidisciplinary collaboration (teamwork), an honest and empathetic communication between the early palliative care team, the patient, and the caregiver (rapport building), and the ability to detect changes in the physical/psychosocial wellbeing of the patient, along the whole disease trajectory (constant monitoring).

**Methods:**

This community case study documents the quantitative and qualitative results of a long term clinical and research experience in delivering early palliative care service to address both solid and blood cancer patients' and their primary caregivers' needs.

**Results:**

Data showed decreased use of chemotherapy, blood transfusions and referral to intensive care units near the end of life; increased life expectancy; improved symptom burden and mood; increased frequency of goals-of-care and advanced care planning conversations. Hope perception among bereaved caregivers was associated with resilience and realistic expectations raising from honest communication with the early palliative care team and appreciation toward the model. Patients and caregivers perceived the possibility of a good death as realistic and not as an unlikely event as it was for patients and caregivers on standard oncologic care only. Gratitude expressions toward the model and the team were frequently identified in their reports and positively associated with communication and spirituality.

**Conclusions:**

These findings are discussed in the context of an updated literature review regarding value-based care and suggest that early palliative care integrated into standard oncology care may be considered as an effective model of value-based care.

## 1. Introduction

With the advances in oncologic treatments, life expectancy for patients diagnosed with cancer has increased (1); however, advanced solid malignancies and high-risk hematologic neoplasia remain largely incurable. In such a wide clinical scenario, physicians have to face a broad range of patients' needs including those associated with the side effects of the new drugs, those associated with the survivorship and those related to the management of incurable cancers (2–4).

A large body of evidence attempted to assess and address needs in patients with cancer. However, implementation of effective interventions has been suboptimal and unmet needs still represent a challenge in oncology and even more in hematology (5).

Mismatched care models that are inconsistent with patients' and caregivers' needs can potentially lead to poor clinical outcomes such as higher morbidity and mortality and reduced quality of life (QOL) as well as a high healthcare use and expenditure (6–8). This is becoming increasingly incompatible with the call for the adoption of value-based models of care.

Value-based healthcare proposes the combination of medical skills with patients' values to obtain the best outcome at the lowest cost. It combines the highest level of technical-scientific data (technical value) with patient preferences, concerns, expectations, and influences (personal value) and the use of resources in order to obtain the greatest advantage for the population (allocation value) (9, 10).

Ideally, a value-based model of oncology care should assess unmet needs with a flexible approach. Indeed, cancer-related symptoms and patients' needs fluctuate along the whole disease trajectory (4, 11, 12) and in relation to different solid and blood tumor types (13–17).

The unmet needs of patients can increase the level of caregiver burden (4, 18), leading caregivers themselves to experience unmet needs. Caregivers' unmet needs do not only decrease their own QOL, but also affect patients' health outcomes negatively (19–21).

In this scenario, a “paradox” related to the value-based models of care emerges in relation to the situation of patients with incurable oncologic or hematologic cancers: if value is determined by the proportion between health outcomes and the resources used to obtain them, how can value be defined when the obvious outcome is death and not the recovery of the patient?

A number of studies have described and identified connections between the concept of quality of care, QOL and, even, “quality of death and dying” [e.g., (22–28)]. The application of the questionnaire on the “Quality of Death and Dying” (QODD) by Curtis et al. (23) found relevant correlations between the highest QODD scores and factors such as dying at home, lower symptom burden, better symptom management, better communication with the healthcare team, improved satisfaction with treatments.

More recently, an innovative model of assistance, consisting of the integration of palliative care to standard oncological care (SOC) since the diagnosis of incurable cancers, has resulted in improved physical and psychological symptoms, QOL and, even, QOL at end of life (QOL-EOL), suggesting a long-term benefit from interdisciplinary early palliative care (EPC) on care throughout the illness (26).

We claim that in oncology, the integration of EPC to the SOC may represent a value-based model. EPC includes anticipated guidance about symptom management and thoughtful discussions on goals of care that engage individuals to consider their values and care preferences in a more patient-centered and less disease-centered environment than the standard oncologic care (29).

In the following sections, the paper documents the experience of delivering EPC to solid cancer and blood cancer patients in two outpatient clinics in Italy. This description argues that EPC treatments can be considered as a form of value-based care in oncology and puts forward the hypothesis that EPC interventions could actually favor the combination of QOL, quality of care along all the disease trajectory, and the quality of death.

## 2. Context in which the innovation occurs

The provision of EPC described in this paper takes place in two EPC units.

The first is located at the Oncology and Palliative Care Unit of the Civil Hospital in Carpi, within the Local Health Unit in Modena; the second, at the EPC clinic of the section of Hematology, Azienda Ospedaliero Universitaria Policlinico, University of Modena and Reggio Emilia.

In both units, the EPC program involves assessment and management of symptoms, support in decision making and future planning, facilitation of coping and providing physical and emotional support through periodic tutorial meetings with oncologists/hematologists and nurses, as well as the assessment of patients' prognostic awareness, which is considered a crucial element defining an EPC intervention (30).

Patients commonly admitted at the Carpi Unit have advanced solid cancer, i.e., distant metastases, late-stage disease and/or a prognosis of 6–24 months (31). In Modena, patients have mostly acute myeloid leukemia (AML) or multiple myeloma, but also patients with other high-risk hematologic malignancies receive EPC. In both cases, the intervention is defined as “early” when provided within 8 weeks from cancer diagnosis (31–33).

## 3. Detail to understand key programmatic elements

Despite the fact that it is not possible to identify a single reference model that explains all the possible EPC interventions and how they should be implemented (34), the overall structure of an EPC intervention has been described and summarized in a way that shows its main components and their rationale (33, 35–37).

The crucial components of this model can be summarized with three keywords: teamwork, rapport building, constant monitoring. As for “teamwork,” this keyword refers to the style of care characterizing the collaboration between the SOC team and the EPC team: in this model, the two teams never stop cooperating. It also refers to the kind of work developed by the EPC team with all the other physicians and subspecialists involved in patients' care, in addition to other interdisciplinary team members that may be consulted if appropriate (e.g., social worker, spiritual care worker, occupational therapist, physiotherapist, etc.). Finally, it is the EPC team who involves the home-based services when discontinuation of disease-directed care is decided and routine oncology follow-ups cease.

The second keyword, “rapport building,” is a complement to “teamwork” and, in a way, its precondition: rapport building between the EPC team, patients and their families is begun early on, at the very first encounter, during which focus is placed especially on coping and support. The team explores patients' and caregivers' understanding and expectations regarding the disease and palliative treatments; at this point, caregivers' needs are also addressed. The style of care in the EPC clinic aims at maintaining a supportive therapeutic atmosphere and building on rapport established during previous encounters. Thanks to this style of care, over time it is possible to progressively develop discussions about end of life (EOL) and resuscitation status, including in the discussion also patients' family members. Appropriate communication is clearly a fundamental ingredient for “rapport building.”

“Constant monitoring” is at the same time possible because of rapport building and another one of its ingredients. Indeed, as previously mentioned, the needs of advanced cancer patients may change rapidly and the care team must be ready to assess them and decide appropriate interventions. The EPC intervention may entail from three to five visits in order to be considered completed, focusing on symptom management, coping, prognostic awareness, decision-making and EOL planning (35). A key element in EPC interventions is the assessment of pain and other relevant symptoms and coping abilities, which should occur frequently if not at every visit. Moreover, if the minimum for a complete EPC intervention amounts to at least 1 monthly visit for the first 4 months, it is true that after the first visit the care team and patients/caregivers remain in constant contact, in order to manage sudden needs or symptoms, thus avoiding unnecessary visits to the clinic or to the ER.

### 3.1. The interventions in Carpi and Modena

The EPC units in Carpi and Modena operate largely based on the model described by Zimmermann et al. (33) and Greer et al. (35). In particular, as far as the unit in Carpi is concerned, a retrospective observational study observed different clinical indicators for 292 advanced cancer patients consecutively admitted at the Unit between 2014 and 2017 and with at least three or more palliative care visits from the time of diagnosis (31). Patients were assigned to either “early palliative/supportive care” or “delayed palliative/supportive care” groups, based on the time elapsed between the diagnosis and the initiation of the palliative care, using 90 and 60 days as a cut-off in a primary and secondary analysis, respectively.

The study confirmed a favorable association between EPC intervention and the index of EOL aggressiveness represented by the administration of chemotherapy in the last 14, 30, and 60 days of life, respectively. Specifically, the frequency of chemotherapy use in the last 60 days of life was 3.4% in the early group and 24.6% in the delayed group. This result is in line with similar results reported in the literature (29, 38) and seems to be strongly favored by improved patient prognostic understanding and shared decision-making, especially in the phase of transitioning from disease-directed care to supportive care alone. Other relevant findings of this study are that patients with advanced cancer enrolled in an EPC program were likely to experience an increase in their survival length, with an estimated survival probability at 1 year of 74.5% in the early group and 45.5% in the delayed group, and - regardless of the timing of palliative care referral - were more likely to have home deaths, and were more likely to report improved symptom burden and mood, as assessed by the Edmonton Symptom Assessment Scale.

A similar observational, retrospective study was conducted at the Modena Unit, aiming to investigate the presence of quality indicators for palliative and EOL care on 215 patients affected by acute myeloid leukemia. All patients were on palliative care, which was defined early when patients received three or more visits or delayed when patients received only one or two visits. Patients with acute promyelocytic leukemia and those undergoing allogeneic hematopoietic stem cell transplantation were excluded. Indicators were abstracted through a comprehensive review of their hospital chart (32). The results are similar to those of the Carpi study: very few patients (2.7%) received chemotherapy in the last 14 days of life; none of them was admitted in the intensive care unit during the last month of life; approximately half of them (50.7%) died at home or in a hospice vs. 5.3% who died in an acute facility; more than 40% received either red cell (49.3%) nor platelet (41.3%) transfusions within 7 days of death. More than 70% (71.8%) of patients receiving EPC had goals of care discussions, and almost 60% (57.3%) had advance care planning conversations.

In relation to the interventions in Carpi and Modena, there are other three studies worth mentioning because they further explore benefits deriving from the EPC interventions as implemented in these two units. More specifically, these studies explore the perceptions of hope and death and the emergence of gratitude in patients and caregivers recruited in both units between July 2020 and June 2022. Patients involved in the studies had advanced cancer whereas caregivers had an alive and/or a deceased patient with advanced cancer. Their eligibility required at least four visits at the EPC unit, willingness to complete the task, and age ≥18 years. At the time of the enrollment, patients had a life expectancy of more than 6 months and were not on interim evaluations to be referred to hospice or home care. The relevance of these studies is explained by the fact that the way patients and caregivers perceive hope and death, as well as the positive emotions arising, although unsolicited, after the EPC intervention, can make a huge difference on their QOL and quality of death and dying; moreover, there is a substantial lack of studies exploring these dimensions qualitatively and based on patients' and caregivers' perceptions (26, 39).

In the first study, hope perceptions among bereaved caregivers of onco-hematologic patients who received EPC were explored (40). The participants of this study were 36 primary caregivers (14 males, 22 females) of deceased onco-hematologic patients treated with EPC at the Carpi Unit (*n* = 26, caregivers of solid tumor patients) and at the Modena Unit (*n* = 10, caregivers of hematologic tumor patients). Open-ended questionnaires asking about caregivers' experience with EPC were administered to participants, 2 months to 3 years after a patient death. Definitions of hope in the caregivers' narratives were analyzed through a directed approach to content analysis (41), which is one of the best-known methods to conduct qualitative research in the medical sciences on textual data, often adopted when there exists research on a certain phenomenon. The Based on the coding categories identified in the existing literature, which capture the main functions of hope (i.e., hope as expectation, hope as resilience, hope as desire), the main results of this study show that caregivers perceived hope mainly as resilience and as expectations based on what they were told about the patients' clinical conditions. Their hope was bolstered by trusting relationships with the healthcare teams and EPC interventions were recalled as the major support for hope, both during the illness and after the death of the patient. Results were complemented with automated lexicographic analysis on the words “hope” and “desire,” to characterize their use in primary caregivers' definition of hope versus its meaning in everyday use, by identifying their relevant combinatorial properties, i.e., their recurrence with adjectives, adverbs and prepositional phrases. The automated quantitative lexical analysis provided deeper insights into the links between the concepts of hope, truth, and trust, which, in the respondents' words, form a tight semantic cluster. These findings suggest that telling the truth about an incurable onco-hematologic disease and beginning EPC might be a combination of factors fostering the onset of hope in the setting of incurable cancer.

In the second study, perceptions of death among patients with advanced cancer receiving EPC and their caregivers were explored, following a mixed method analysis (42). In this case, qualitative and quantitative analyses (43–45) were performed on two databases: (a) transcripts of open-ended questionnaires investigating thoughts and feelings about the personal experience with the disease prior and during the EPC intervention and about possible changes in the perception and expectations of their future administered to 130 cancer patients receiving EPC, and to 115 primary caregivers of patients on EPC treated in the two above mentioned units; (b) texts collected from an Italian forum, containing instances of web-mediated interactions between patients and their caregivers. The quantitative analysis consisted of extracting the combinatorial properties of the word “death” from the two databases and representing the most frequent combinations of words by means of Sketch Engine, a platform commonly used by linguists, translators, and lexicographers to analyze the meaning of lexical entities through text mining functions. The qualitative analysis was performed on the combinatorial properties by considering the semantic context in which they appeared, with the aim to provide context for the interpretation of these results. The most interesting finding in this study shows that for patients and caregivers on EPC the word “death” has positive and actual connotations, i.e., it expresses an experience, whereas for the participants interacting on the forum, a “good death” is referred to as a wish or as a negated event. These findings suggest that EPC interventions may be among the factors that favor an increased acceptance of death among advanced cancer patients and their caregivers.

In the third study, the hypothesis that a feeling of gratitude might be commonly encountered among cancer patients and their caregivers on EPC was explored (39). Reports from 251 patients with advanced cancer on EPC (*N* = 133; 73 males, 60 female) and their caregivers (*N* = 118; 39 males, 77 females) describing their clinical experience with the EPC model were analyzed through a content analysis and a quantitative text analysis program, to identify and rank the sources of gratitude and to quantify the use of words associated to categories of interest (i.e., gratitude, communication, spirituality), respectively. The presence of explicit or implicit expressions of gratitude were found in most of the reports (92.5% and 82.2% for patients and caregivers, respectively). Moreover, the identified sources of gratitude were structural components of the EPC intervention, namely: successful physical symptom management (mentioned by 83.5% of patients and 78% of caregivers), emotional support (mentioned by 46.6% of patients and 39% of caregivers), empowerment from the conversations on EOL (mentioned by 33.8% of patients and 11% of caregivers), better information (mentioned by 24.1% of patients and 22% of caregivers), humanity (mentioned by 24.1% of patients and 22% of caregivers), and a familiar environment (mentioned by 12% of patients and 14.4% of caregivers). Finally, the emergence of gratitude in patients' reports was positively associated with references to communication with the palliative team (*r* = 0.215, *p* = 0.026) as well as to spirituality (*r* = 0.612, *p* < 0.001). These results suggest that EPC and the associated benefits would unintentionally elicit positive emotions that, based on the positive psychological wellbeing (46), may represent useful resources for patients and caregivers, as well as a potent predictor of improved health outcome. Of note, in all the aforementioned studies a certain style of communication appears in connection with the benefits deriving from EPC interventions.

Another relevant and unique characteristic of the interventions in Carpi and Modena is that the mean number of EPC visits is significantly higher than those of three to five reported in literature, strongly suggesting that patients are conducted along the entire disease trajectory.

Indeed, several cohort studies have reported that inpatient PC, by fostering death at home, increases QODD. Nonetheless, in a secondary analysis of a cluster-randomized trial of EPC in advanced solid cancer patients (47), there was no association between EPC and overall QODD and QOL-EOL, and EPC exerted a significant and large effect also on QOL-EOL only when additional palliative care were added along the trajectory of the disease (26).

Thus, by managing invalidating symptoms, cultivating the prognostic awareness, favoring patients' and caregivers' understanding of treatment progress, helping with decision-making, exploring patients' values and assisting in the promotion of advanced care planning, in Carpi and Modena EPC positively affects patients' and caregivers' QOL and, by providing support along the entire trajectory of cancer, fosters “quality of death and dying” for patients and their caregivers.

## 4. Discussion

Value-based healthcare is a relatively new approach, which “aims to increase the value that is derived from the resources available for a population” (48, 49). However, there is not yet a complete consensus among scholars regarding what should be considered “value” in healthcare (10, 49–51).

Moreover, from various studies that observed cases of implementation of the value-based healthcare model, it is emerging that a crucial factor, albeit the less measurable one, is the quality of information production and circulation among all the stakeholders involved in the creation, provision and assessment of healthcare. Indeed, the dissemination of a “value culture” (52) can only happen *via* effective education, which involves sharing information about value and how to obtain it. Also the major tenet of value-based healthcare–i.e., the consideration of which outcomes are relevant for patients (53)–implies taking into consideration patients' views and preferences, which again involves effective communication strategies. In particular, the stress on patient-centeredness and on patient involvement is probably the major strength and at the same time the major challenge for the implementation of value-based healthcare, because personal perceptions and preferences by definition fluctuate and are not easily formalized in the way that would be required by an effective managerial model; indeed, various studies highlight the fact that value based healthcare can only be effectively implemented if the whole system accepts to be redesigned according to the concept of “value” (51, 54, 55).

In this sense, the EPC model could be considered as an example of successful value-based healthcare provision. The provision of care in an EPC model necessarily implies spending time with patients and their families in order to: build the kind of relationship that will allow addressing difficult topics; understand patients' and caregivers' clinical needs; understand patients' and caregivers' psycho-social or spiritual needs that have an import on their wellbeing (33, 36, 37, 56).

Moreover, regarding the feasibility of value-based healthcare, scholars have identified six interdependent and mutually reinforcing steps toward a high-value healthcare delivery system (52, 57–59). These are: 1. Organize integrated practice units; 2. Measure costs and outcomes for every patient; 3. Move to bundled payment for the care cycle; 4. Integrate care delivery across separate facilities; 5. Expand excellent services across geography; 6. Enable a suitable information technology platform. The EPC model of care seems to satisfy at least four of these steps: in order to be called an EPC intervention, it requires that different units of practice are integrated, and it is able to integrate care delivery across separate facilities, for example when transitioning from disease-oriented care to home care (33) (points 1 and 4); it has also been shown to be a cost-effective model (60–63), although there are still few studies based on sufficiently big samples. Indeed, adopting value-based care supports health care providers in their decisions while focusing on the values of patients, leading to lower healthcare costs, regardless of professionals' concern with the cost of treatment (64) (point 2). As regards point 6, there is mounting evidence that digital health technology, in the form of platforms allowing the electronic collection of patient reported outcomes (PROs), can have a positive impact on the overall management of cancer patients. Indeed, two recent RCTs in patients with several types of cancer during chemotherapy showed that remote symptom monitoring with electronic PROs was associated with reduced symptom burden and improved HRQoL outcomes (65, 66). Remarkably, the systematic monitoring of PROs *via* web-based platforms, was also found to be associated with improved overall survival in patients with advanced cancers (67, 68). Finally, a study examining physicians' perceptions of usability and clinical utility of a digital health tool (GIMEMA-ALLIANCE platform) for ePRO monitoring in the real-life practice of patients with hematologic malignancies found that all hematologists participating in the study agreed or strongly agreed that the platform was easy to use, and 87%, agreed or strongly agreed that ePROs data were useful to enhance communication with their patients (69). These preliminary results support the clinical utility, from the perspectives of the treating hematologist, of integrating ePROs into routine cancer care of patients with hematologic malignancies, and could be implemented in the EPC interventions.

With regard to the specific meaning of “value” involved in the treatment of advanced/high risk cancer patients and their families, we suggest that EPC treatments may also be successful in achieving the three levels of quality described by Curtis et al. (23). QOL-EOL has been shown to be associated with a systematic use of integrated palliative care (70) and is mostly associated to lower or no use of palliative chemotherapy, which has been shown to worsen patients' QOL and quality of death (71, 72). Aggressive treatments at the EOL are also usually considered as signs of low quality of care (73–75); the integration of EPC has been shown to reduce aggressive measures at the EOL, thus promoting quality of care (75–81).

As for the quality of the dying experience, the analysis of responses to questionnaires about perceptions of hope and death at the EPC Units in Carpi and Modena testify to perceptions of high quality. In the future, these should be verified also by the use of the QODD questionnaire.

Although the kind of value that needs to be obtained in an EPC setting (QODD) may be different from the one that is called for in other clinical settings (mainly QOL), a certain approach to care could be used as a model to progressively implement a value-based model of care along the entire trajectory of the disease.

Future research in this area should also focus on grounding the EPC model in a theoretical frame. Each intervention provided in the EPC context and described in this work arises from a large amounts of empirical, real-life data, in a bottom-up fashion. However, its robustness and validity require to be supported and further confirmed also through a top-down approach in order to define a univocal model whose use can be extended to different onco-hematology populations. This would be beneficial to the model, also in terms of the flexibility required to support different types and different stages of the disease, but also to be extended to most medical specialties dealing with serious illnesses and close to the EOL.

## 5. Acknowledgment of conceptual or methodological constraints

We acknowledge that the model described in this article may be difficult to implement due to a few conceptual and methodological constraints.

As for conceptual constraints, it has been observed that the integrated EPC model of care has been described only in a standardized form, thus leaving it to professionals to devise specific strategies that will allow its implementation in local systems (33).

In a methodological perspective, a significant constraint is represented by the limited awareness still observable in the population regarding the existence of EPC clinics; moreover, oncologists' hesitancy to refer patients to palliative care and specific training for clinicians may also hinder the implementation of the proposed model of care (82, 83).

Regarding the situation in Italy, where the case study described in this article was developed: of note, following the conversion of the law decree of May 19, 2020 into law, the Specialty School in Medicine and palliative care has been created (https://www.gazzettaufficiale.it/eli/gu/2020/08/31/216/sg/pdf), beginning in the academic year 2021–2022 (84). A more structured and comprehensive training of professionals in palliative care will hopefully facilitate the adoption and optimal implementation of the model. A clear training pathway as dual board-certified medical hematologist/oncologist and (early) palliative care physician is worth pursuing, in order to avoid hematologists and oncologists still confusing palliative care with end-of-life care (85, 86).

## Data availability statement

The original contributions presented in the study are included in the article/supplementary material, further inquiries can be directed to the corresponding author.

## Author contributions

All authors contributed to conception and design, bibliography review and data analysis and interpretation, manuscript writing, and final approval of manuscript.
